# 3D Printing of Physical Organ Models: Recent Developments and Challenges

**DOI:** 10.1002/advs.202101394

**Published:** 2021-07-08

**Authors:** Zhongboyu Jin, Yuanrong Li, Kang Yu, Linxiang Liu, Jianzhong Fu, Xinhua Yao, Aiguo Zhang, Yong He

**Affiliations:** ^1^ State Key Laboratory of Fluid Power and Mechatronic Systems School of Mechanical Engineering Zhejiang University Hangzhou Zhejiang 310027 China; ^2^ Key Laboratory of 3D Printing Process and Equipment of Zhejiang Province School of Mechanical Engineering Zhejiang University Hangzhou Zhejiang 310027 China; ^3^ Zhejiang University Hospital Zhejiang University Hangzhou Zhejiang 310027 China; ^4^ Department of Orthopedics Wuxi Children's Hospital affiliated to Nanjing Medical University Wuxi Jiangsu 214023 China; ^5^ Key Laboratory of Materials Processing and Mold Zhengzhou University Zhengzhou 450002 China

**Keywords:** 3D printing, physical organ models, polymer materials, surgical applications

## Abstract

Physical organ models are the objects that replicate the patient‐specific anatomy and have played important roles in modern medical diagnosis and disease treatment. 3D printing, as a powerful multi‐function manufacturing technology, breaks the limitations of traditional methods and provides a great potential for manufacturing organ models. However, the clinical application of organ model is still in small scale, facing the challenges including high cost, poor mimicking performance and insufficient accuracy. In this review, the mainstream 3D printing technologies are introduced, and the existing manufacturing methods are divided into "directly printing" and "indirectly printing", with an emphasis on choosing suitable techniques and materials. This review also summarizes the ideas to address these challenges and focuses on three points: 1) what are the characteristics and requirements of organ models in different application scenarios, 2) how to choose the suitable 3D printing methods and materials according to different application categories, and 3) how to reduce the cost of organ models and make the process simple and convenient. Moreover, the state‐of‐the‐art in organ models are summarized and the contribution of 3D printed organ models to various surgical procedures is highlighted. Finally, current limitations, evaluation criteria and future perspectives for this emerging area are discussed.

## Graphical abstract

3D printing technologies allows simple and convenient manufacturing of multifunctional organ models, which has been an emerging direction of 3D printing in medicine. Herein, the current methods and recent advancements in 3D printing of organ models are reviewed, with focus on improving the performance and cost effectiveness. The evaluation and future development of 3D printing of organ models are discussed.

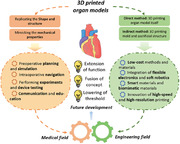

## 1 Introduction

In the clinical treatment of many diseases, how to better simulate and represent the disease states is the content that people always care about. Simple models to simulate the anatomy and pathological properties of organs have been used in medicine for centuries, such as clay and stone models of humans were used to demonstrate clinical features of disease.^[^ ^1^ ^]^ Along with the development of medical image acquisition and processing technology, more and more high‐resolution noninvasive imaging techniques allow to generate more detailed characteristics of patient's organs.^[^ ^2^ ^]^ Doctors use these images to gain insight into pathology^[^ ^3^ ^]^ and help diagnose disease, but the limited representation of three‐dimensional (3D) anatomy by two‐dimensional (2D) images may result in the loss of details of inter‐tissue relationships. Virtual 3D models, while improving these conditions to some extent, do not provide realistic tactile properties, nor do they allow doctors to operate in reality. To ameliorate these circumstances, making a physical model of a patient's specific organ is expected to come to the aid.

3D printing, also known as additive manufacturing (AM), is a rapidly developing manufacturing technology, has gradually become an emerging and crucial adjunctive tool in medicine,^[^ ^4^ ^]^ and is the preferred method for fabrication of organ models. 3D printing constructs complex functional 3D structures layer by layer from various materials,^[^ ^5^, ^6^, ^7^ ^]^ and makes the data from computer‐aided‐design (CAD) modeling or scanning be converted into a 3D physical model.

Unlike conventional manufacturing process that involves milling and cutting of pieces or even hand‐made molds to build the structures, 3D printing allows rapid fabrication of various structures, such as the shapes of organs and blood vessels, with almost no waste of excess material. Coupled with the constant innovation of different principles and the gradual reduction of the cost of 3D printer and materials, 3D printing has become a low threshold, versatile advanced manufacturing methods, meeting the growing application needs in a number of fields.

In recent years, with the development of 3D printing technology, applications of organ models have also undergone a revolutionary progress. A growing number of interdisciplinary researchers, doctors and engineers from diverse backgrounds are collaborating to create different kinds of organ models personalized to each patient. Organ models made by 3D printing not only replicate the characteristics, but also approach the physical properties of real organs, and therefore they assist surgeons for presurgical and intraoperative work. Doctors use models to simulate operations, train skills and communicate better with patients before the surgery, and for navigation during the surgery to reduce the duration and risk. Beyond surgery, models could be used to perform repeatable experiments, test medical equipment, or be taken into classrooms to improve medical students’ understanding of knowledge. Furthermore, organ models facilitate exchange and cooperation between medicine and engineering, and promote the development of additive manufacturing in medicine, including artificial organs and biological 3D printing. In the near future, 3D printing of organ models will still be a hot topic that presents great potential to promote the cause of human life and health.

However, 3D printed organ models still remain in the stage of small‐scale application, and existing studies reported some major challenges for further use are: 1) High cost, including time and expenses. Some commercial 3D printers, materials and necessary software are very expensive, while some complicated image segmentation and model creating processes result in several days of labor and require a long learning period.^[^ ^8^, ^9^, ^10^ ^]^ 2) Limited simulation characteristic. It is still difficult for current materials to perfectly mimic the soft tissue.^[^ ^3^ ^]^ A few soft materials that mimic well but are not suitable for direct 3D printing. 3) Low production accuracy. The accuracy is mainly affected by the resolution of imaging and 3D printing. At the same time, the available printing space of a 3D printer is limited.^[^ ^11^ ^]^ Large models may need to be divided into several parts for printing and then assembly, which will also make errors.

**Figure 1:**
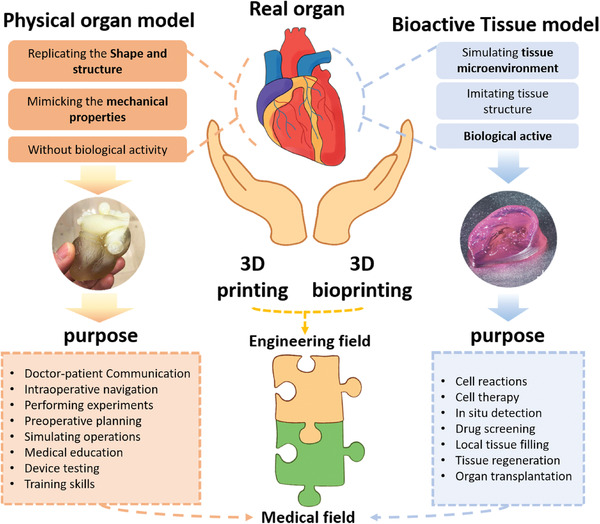
The difference between organ model and tissue model (e.g., tissue/organ construct. Reproduced with permission.^[^ ^15^ ^]^ Copyright 2019, IOP Publishing), where 3D printing and 3D bioprinting play their respective roles.

This review aims to discuss the recent advances in the 3D printing of organ models and provide insights into the better use of this technology under manufacturing constraints in a medical instead of industrial environment. Here, we define an organ model as a physical model that replicates the patient‐specific anatomy to help with clinical treatment or education, but it is not biologically active and is not implanted into human body, which is different from tissue model,^[^ ^12^, ^13^, ^14^ ^]^ and implant (**Figure** **1**).^[^ ^11^ ^]^ We focus on three points: 1) What are the characteristics and requirements of organ models in different application scenarios; 2) How to choose the suitable 3D printing methods and materials according to different application categories? 3) How to reduce the cost of organ models and make the process more simple and convenient?

Based on the above, in this review, we define the physical organ model, and then introduce the mainstream 3D printing technologies and available materials, emphasize on the process and characteristics of each method, because for the specific environment, reasonable selection of technology is an important means to reduce the cost. Then we discuss the feasibility of organ models in clinical application, including the major surgery, prostheses as well as repeatable physiological research. Next, we emphatically discuss the advantages and limitations of 3D printed organ models in these conditions. Finally, we presented our perspective on the future directions.

## 2 Overview of 3D Printing Technologies

3D printing technology is a rapid prototyping method that directly produces 3D objects from CAD data in computer. It is a comprehensive technology that combines the computer aided design (CAD), computer numerical control (CNC), mechanical technology and material science, and adopts the basic method of layered overlay. The CAD model is divided according to a certain layer thickness, and then printed by 3D printers with different functions using specific materials. The nozzle or optical devices are driven by the computer to form a layer of structure on the substrate at a time, and the complete object is constructed layer‐by‐layer. Such method of adding materials is the main difference between 3D printing and the traditional method known as subtractive manufacturing.

In the 1980s the first 3D printing technique called Stereolithography (SL) pioneered by Charles Hull, and was used in its early days as a rapid prototyping process in industrial applications such as aerospace and automobiles.^[^ ^16^ ^]^ In the subsequent development of 3D printing, various new printing techniques and materials have been developed. Nowadays, there are dozens of known 3D printing processes,^[^ ^17^ ^]^ and the materials used cover a wide range of metals, polymers and biological materials. According to different principles, 3D printing technologies are generally classified into seven categories:^[^ ^6^ ^]^ vat photopolymerization, material extrusion, material jetting, powder bed fusion, binder jetting, sheet lamination, and energy deposition.

Not all 3D printing technologies are suitable for creating organ models. Considering the principle of different techniques, available materials, performance and cost, the first five techniques are ideal choices. In particular, vat photopolymerization, material extrusion and material jetting are the most commonly used techniques in 3D printing of organ models, which mainly take the form of a liquid solidification or melting of solid.^[^ ^18^ ^]^ Powder bed fusion and binder jetting, which are mainly based on the combination of powders, also play a role in a number of related applications. In contrast, for the last two techniques, Sheet lamination mainly binds sheet materials such as paper to form an object, and energy deposition, on the other hand, uses focused thermal energy to melt and deposit metals directly in a specific location. Due to the properties of paper and metals used in these two techniques, it is not suitable for them to make organ models in most cases. In this section, we will introduce these five commonly used printing technologies, and a comprehensive comparison of them is shown in **Table** **1**.

**Table 1:** Comparison of different 3D printing technologies

Categories^a)^	Typical materials	State and form of material base	Approximate maximum resolution	Cost^[^ ^9^, ^18^ ^]^	Common 3D printers
Vat photopolymerization	Resin	Liquid	5 µm^[^ ^20^ ^]^	++	ProJet 7000 HD (3D Systems) Lite 300 3D printer (UnionTech) Form 2/ 3 (Formlabs) JGMaker G3 (JGMaker)
Material jetting	Resin	Liquid, droplets	10 µm^[^ ^20^ ^]^	+++	OBJET 260/350/500 Connex 3 (Stratasys) J750 (Stratasys) J401 (Sailner 3D)
Material extrusion (e.g., FDM)	Thermoplastics	Solid, filament	100 µm^[^ ^20^ ^]^	+	Makerbot x2/ Method (MakerBot Industries) Ultimaker 2+/ S3 (Ultimaker) STRATASYS F370 (Stratasys) UP plus2 (Tiertime)
Powder bed fusion	Nylon, metal	Solid, powder	100 µm^[^ ^20^ ^]^	+++	LaserCore5300 (Longyuan AFS) Fuse 1 (Formlabs)
Binder jetting	Polymer, plaster	Solid, powder	90 µm^[^ ^51^ ^]^	+	ProJet CJP 460Plus/ ProJet 660 Pro (3D Systems) Z printer 450/ 650 (3D Systems) Spectrum Z510 (ZCorp)

^a)^The cost of 3D printing is affected by many factors, such as filling patterns, filling density, and model orientation,^[^^9^^]^ so here a qualitative comparison of cost is provided, where more “+” means higher cost and same number of “+” only means they have similar cost.

### 2.1 Vat Photopolymerization

Vat photopolymerization is a process in which photosensitive polymer liquid materials are cured layer by layer using a specific light to form an entity. Materials used in this technique can also be collectively called as photopolymers, which will change their state from liquid to solid when exposed to specific light source (e.g., UV light). The requirements of good liquidity of photopolymers ensure that these materials evenly spread in the printing area, and the optical system in the 3D printer controlled by the computer ensures only the required area is hardened. Polymers with epoxy based and hybrid systems are well received due to their higher temperature resistance, lower moisture absorption and shrinkage compared with acrylate based materials.^[^ ^19^ ^]^ In addition, elastic resins, silicone elastomers and hydrogels are also seen to be used for vat photopolymerization.^[^ ^20^ ^]^

**Figure 2:**
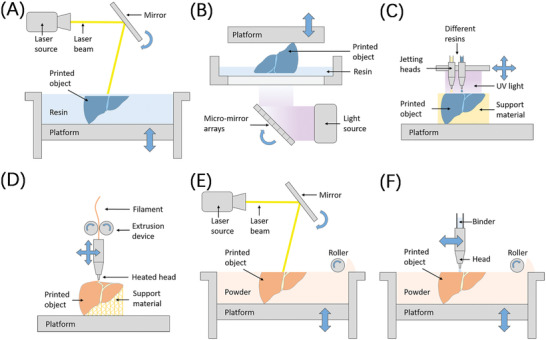
Schematic illustrations of different 3D printing technologies. A) Stereolithography (SLA). B) Digital light processing (DLP). C) Material jetting (e.g., Polyjet printing). D) Material extrusion (e.g., FDM printing). E) Power bed fusion. F) Binder jetting.

Stereolithography (SLA) (**Figure** **2**A) was the first vat photopolymerization technology and the first 3D printing technology,^[^ ^16^ ^]^ and remains a widely used method today. Since it advent, based on the concept of photopolymerization, some new methods have been developed, such as the technique using micro mirror arrays called digital light processing (DLP)^[^ ^21^ ^]^ (Figure 2B) has been seen in many laboratories, as well as the further method microstereolithography (MSL),^[^ ^22^ ^]^ continuous liquid interface production (CLIP),^[^ ^23^ ^]^ and computed axial lithography (CAL),^[^ ^24^ ^]^ have made a qualitative leap in the printing speed and the resolution, but there is still a lack of large‐scale application in industrial production.

Photopolymerization methods have relatively higher manufacturing accuracy compared with other 3D printing techniques. Furthermore, benefiting from their principle of 2D scanning, the DLP, CLIP, and CAL techniques have a marked advantage in printing speed. But the materials used may be expensive, and the multimaterial ability of photopolymerization methods is always limited due to the residue of materials on the surface of 3D printed structure.

### 2.2 Material Jetting

Material jetting is another 3D printing technology based on liquid curing, also known as inkjet printing. Unlike vat photopolymerization, the print material is ejected through a print nozzle as a droplet or liquid jet. Droplets are deposited and rapidly cured by an auxiliary curing device (usually by UV light) to build each layer of patterned structure, as well as provide a high print resolution. Drop‐on‐demand (DOD) is one of the typical modes of inkjet printing. In this mode, the printer does not produce droplets continuously, but only when needed. The droplet producing process of the ink used in inkjet printing depends on many factors, including printing parameters and fluid properties. Fluid density (*ρ*), dynamic viscosity (*η*), surface tension (*γ*), characteristic droplet length (*l*), the velocity of the ejected droplet (*v*), and the nozzle diameter (*d*) are all the parameters that affect this process.^[^ ^20^ ^]^ Meanwhile, the requirements of rheological properties of printable ink are determined by fluid mechanics of the droplet producing process.^[^ ^25^ ^]^ Suitable ink properties can be expressed by Reynolds numbers (*Re*), Weber numbers (*We*) and Inverse Ohnsorge (1/Oh)

(1a) Re=υρl/η

(1b) $$We=\upsilon^{2}\rho l/\gamma$$

(1c) 1/Oh=Re/We=η/γρl

It has been proved that droplets can be stably formed when 1/Oh is between 1 and 10.^[^ ^25^ ^]^ Otherwise, if 1/Oh is too small, the droplets will be prevented from ejecting by viscous dissipation, and if 1/Oh is too large, the droplets are more likely to splash or break up into satellite droplets. Moreover, the fluid/air surface tension at the nozzle imposes additional limits to droplet generation. To overcome this barrier, there should be *We* > 4, specifically, the velocity of the ejected droplet (*v*) should be larger than (4*γ*/*ρd*)^1/2^.

Polyjet technology (Figure 2C) is one of the representatives of material jetting.^[^ ^26^, ^27^ ^]^ The multiple jetting equipment allows easy control of the composition of the material, enabling material jetting to have powerful multi‐material capabilities and be good at building support structures, or multi‐material construction of different properties and colors without additional assembly steps.^[^ ^20^ ^]^ In addition, it only requires simple post‐processing. These are the key advantages of material jetting. On the other hand, the printing speed of material jetting is similar to SLA, which is enough in most applications. All of these factors make material jetting be widely applied on 3D printing of organ models, but it is still limited to some extent by the high cost of materials and equipment.

### 2.3 Material Extrusion

Material extrusion is a 3D printing technology based on the melting of solid. Similar to selective deposition of materials by material jetting, material extrusion uses a nozzle to extrude filamentous materials to construct structure. Various thermoplastics, such as polylactic acid (PLA), are commonly used in this technique, which is fed into a nozzle and heated to a molten state, then cooled and hardened on the platform after extrusion. Additional printing of support structures is necessary when faced with hollow or large inclined structures. The principle of material extrusion makes it has relatively lower printing speed than most other 3D printing methods.

Fused deposition molding (FDM) (Figure 2D) is the earliest material extrusion method and is popular worldwide arising from its low cost.^[^ ^28^, ^29^ ^]^ To break through the limitation of the rigid materials, the printing method for soft materials in material extrusion has been gradually explored. Direct ink writing (DIW) is a kind of typical method in which the viscoelastic material as the “ink” for printing.^[^ ^30^, ^31^ ^]^ Unlike FDM method based on the melting of materials, DIW printing relies on the specific rheological properties of materials.^[^ ^32^, ^33^ ^]^ Through comprising different kinds or concentrations of additives in the material systems, the rheological properties of the material can be systematically adjusted to achieve two characteristics, shear thinning behavior and good recoverability. Specifically, the dynamic modulus of the material includes the storage modulus (*G’*) and the loss modulus (*G’’*), which respectively reflect the elasticity and viscosity of the material. The intersection of *G’* and *G’’* is defined as the yield point, at which the shear stress is the yield stress, signifying that more energy is dissipated viscously than stored elastically.^[^ ^34^ ^]^ Therefore, the shear stress at the nozzle exerted by the mechanical pressure exceeds the yield stress of the ink makes the ink fluidize and mainly undergoes viscous deformation (*G’* < *G’’*), which determines the proper printability window. When the ink is extruded and deposited, it can rapidly recover its initial elasticity and storage modulus (*G’* > *G’’*), exhibits a gel‐like state with very high elastic modulus and viscosity, and thus maintains the shape after printing.^[^ ^35^, ^36^ ^]^

The printable ink for DIW is non Newtonian fluid with a yield stress that can be well described by the Herschel‐Bulkley model:

(2) $$\tau =\tau_{y}+K{\dot{\gamma }}^{n}$$

where *τ* (Pa) is the shear stress applied, *τ*_*y*_ (Pa) is the yield stress, *K* (Pa s*^n^*) is the consistency, $\dot{\gamma }$ (s^–1^) is the shear rate, and n is the flow index (dimensionless coefficient, measuring the degree of the shear thinning or shear thickening of the fluid. For shear thinning fluids, *n* < 1).

The above printing mechanism gives the DIW method excellent ability to print soft materials, such as silicone^[^ ^37^ ^]^ and hydrogels,^[^ ^15^, ^38^, ^39^, ^40^ ^]^ and has played a significant role in biological manufacturing. In the future, it is also expected to be used to make organ models that are more approximate to the properties of soft tissue.

### 2.4 Powder Bed Fusion

Powder bed fusion is a 3D printing process based on powders that uses high power laser or electron beam to melt and bind the material particles at a specific location to create a solid object. Selective laser sintering (SLS) (Figure 2E) is the most common method,^[^ ^41^ ^]^ and similar ones are also selective laser melting (SLM), which can be discussed together. Metal is available for SLS, as well as ceramics, plastics, glass and other granular materials. Powders are laid in a thin layer on a platform at a time. The materials in unsintered regions can provide support and then be recycled, thus the printing process does not require additional support. For printing speed, although SLS technique is a 0D scanning method, the long term development and full investigation of the process parameters and devices enable it to construct structures vary fast and stably. However, a problem is that it is hard for SLS to reach highly surface finish and dimensional accuracy, which often leads to deficient structure and requires more post‐processing work.^[^ ^42^, ^43^ ^]^ The printing resolution basically depends on the powder size, and the material properties are also a valid factor affecting the sintering effect.^[^ ^44^, ^45^ ^]^ In consideration of the high cost of SLS devices and the limitation of available material types,^[^ ^46^ ^]^ few organ models are processed directly by SLS, but are more inclined to make molds by it first.

### 2.5 Binder Jetting

Similar to powder bed fusion and material jetting, binder jetting (Figure 2F) uses both a powder base and a jetting head, but the difference is the utilization of binder substances instead of lasers. The binder agents are selectively jetted to cement powder materials to build 3D structures.^[^ ^47^ ^]^ Binder jetting can use gypsum powder^[^ ^10^, ^48^ ^]^ in addition to the materials available for powder bed fusion. Binder jetting also has a high printing speed, especially, the speed can be further improved by increasing the number of printing nozzles. Besides, the 3D printers of binder jetting are always small, quiet and low‐cost.^[^ ^49^ ^]^ When the method is applied in medical field, it is noteworthy that the glues are often toxic,^[^ ^50^ ^]^ even for organ models that are not biologically active in clinic use will also be affected. Likewise, binder jetting is primarily used for mold fabrication.

## 3 3D Printing of Organ Models

**Figure 3:**
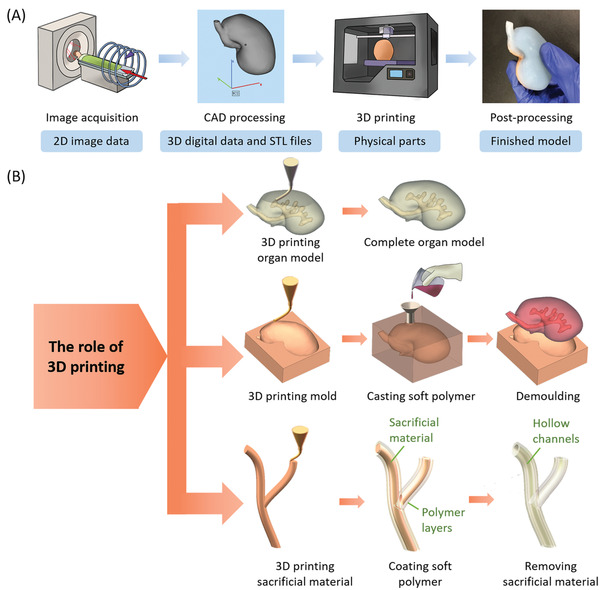
Typical process of 3D printing of organ models. A) The general process of making an organ model and the products of each step. B) The role of 3D printing in different methods, including 3D printing organ model itself, 3D printing mold for casting, and 3D printing the sacrificial material.

3D printing is a powerful manufacturing technology, but it first depends on the appropriate CAD data in the computer before realizing all kinds of amusing ideas. “Appropriate” means that the CAD data is both suitable for 3D printing and a file format acceptable to the 3D printer. To obtain these data, medical images are first acquired through computed tomography (CT), magnetic resonance imaging (MRI) and other imaging technologies, and then CAD models are built after special segmentation, processing and conversion. And then get the real object through 3D printing. Therefore, the general process to generate the final physical organ models from original imaging data can be divided into four steps (**Figure** **3**A): 1) image acquisition, 2) image processing, 3) 3D printing, and 4) post‐processing.

The specific approach of each step depends on the use requirements of the organ model. There are several levels of the requirements, from “watching” to “doing experiments”. Only when the demands are determined, can the specific technology, materials and equipment be selected. We merge the first two steps, image acquisition and image processing, as “pre‐steps”. The purpose of each of the pre‐steps is to provide appropriate data for 3D printing, which mainly involves the knowledge of anatomy. It is often dominated by radiologists, operating on medical equipment and computers rather than 3D printers, and sometimes cooperates with researchers from mechanical backgrounds to get the support of CAD technology. 3D printing is a pivotal step. The existing methods can be classified into two categories: direct 3D printing and indirect 3D printing. The difference is whether the organ model is printed directly or not (Figure 3B). In this section, we introduce the major workflow of 3D printing of organ models, with the focus on the use of direct and indirect methods to create organ models.

### 3.1 Image Acquisition and Image Processing

Medical image acquisition is a process of continuously taking a multitude of 2D images according to the slice thickness set before.^[^ ^18^ ^]^ The technologies today come in a great variety of modalities, and the most commonly used are still CT and MRI, which have strong applicability for most organs and the relative ease of image processing.^[^ ^52^ ^]^ Based on these, the process can also be a combination of multiple methods for some specific organs to gain different forms of data, for instance, echocardiographic images for cardiology models^[^ ^53^, ^54^ ^]^ and cone beam CT and multi‐slice CT contribute to craniomaxillofacial models.^[^ ^55^, ^56^ ^]^ The adjustment of slice thickness in the light of the complexity of the anatomy is clearly warranted.^[^ ^57^ ^]^ Through the above procedures, the original image data, usually in DICOM (Digital Imaging and Communications in Medicine) format, will be obtained and then can be processed.

Image processing is a process of transforming DICOM images into 3D digital models. It is also a broad concept, including image segmentation, computer aided design (CAD) and format conversion. In some studies this process will be subdivided into several steps in detail.^[^ ^18^, ^52^, ^57^, ^58^ ^]^ We briefly summarize them as one step because we are more concerned about the efforts on 3D printing.

Proper CAD data is the basis for 3D printing to play a good effect. The first is image segmentation, which is mainly the extraction of the region that contains the required organ in images. Then the 3D digital model can be generated from the segmented 2D image. Due to the limitation of 3D printing, each technology has some structures that are difficult to construct, and thus the digital model needs to be further processed^[^ ^59^ ^]^ in CAD work according to the selected 3D printing method. Concrete operation includes the removal and trimming of unreasonable structures (such as overlapping or irregular facets) that hinder 3D printing, as well as redundant structures (such as burrs and holes) that affect model features, or the design of special molds.

The file formats that 3D printers can accept are limited to several special 3D dataset files, mainly the Standard Tessellation Language (STL) format^[^ ^60^ ^]^ and some newer formats called Additive Manufacturing File Format (AMF)^[^ ^61^ ^]^ or 3D Manufacturing Format (3MF).^[^ ^62^ ^]^ The model data must be converted into files in these formats before it can be 3D printed.

There are several software can meet the demands of image processing,^[^ ^63^ ^]^ ranging from interactive medical image processing software like Mimics (Materialise), D2P (3D Systems), and CAD model processing software like Magics (Materialise), Geomagic Studio (3D Systems) and SolidWorks (Dassault Systems). In many cases, using multiple software together can integrate different functions.

### 3.2 3D Printing

**Figure 4:**
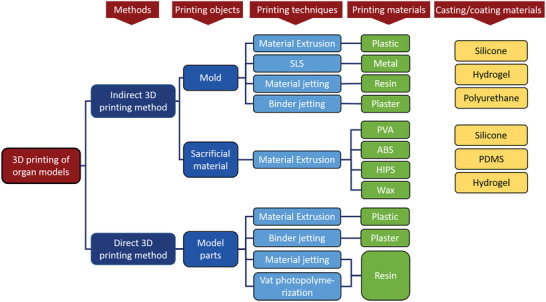
Typical categories of methods and materials for making organ models.

3D printing process can begin when the 3D digital model file prepared in the pre‐step is transmitted to the 3D printer. However, it is often not easy for organ models to be directly 3D printed because of the two inevitable problems: 1) the complex structures of some organ models are difficult to be directly printed, and 2) the existing commercial materials used for 3D printing provide a limited range of hardness, while some soft materials prepared manually are often not suitable for 3D printing.^[^ ^3^ ^]^ Therefore, the priority is to consider in which aspects that let 3D printing work, whether it is directly used for printing organ models, or only used as part of the process of realizing them. The methods of making organ models with 3D printing technology in existing reports can be summarized into these two categories. We define the former as “direct 3D printing method” and the latter as “indirect 3D printing method”. The common technologies and materials are shown in **Figure** **4**. Pietrabissa et al.^[^ ^64^ ^]^ also mentioned these two methods, and we will explain them in detail.

#### 3.2.1 Direct 3D Printing Method

Direct 3D printing method refers to the use of a 3D printer to print the organ model itself directly, but not always print the complete model at one time. The model can also be split into several parts for printing, and then assemble these parts in the post‐processing step. Printing a complete model at one time is obviously the most convenient, but it has restrictions on some conditions. The first is the size. If the proportion of the model is the same as that of the real organ, the size is likely to exceed the maximum printing dimensions of some small desktop 3D printers. The second is the structure. For those 3D printing methods requiring support structures, proper positioning can reduce the support needed for printing small models, but large models often require a mass of support structures that result in the consumption of more materials, longer printing time and post‐processing time. The last is characteristic and appearance. Some full‐featured commercial 3D printers can make organ models using multiple materials and colors at the same time to distinguish different structures,^[^ ^65^ ^]^ while utilizing an inexpensive single‐material 3D printer to print one part of the model at a time or representing specific structures in a more simplified way^[^ ^66^, ^67^ ^]^ are also known to be the ideal methods.

Currently, material jetting, material extrusion and vat photopolymerization are commonly used in direct 3D printing methods. Material jetting, represented by Polyjet technology, is the most popular option among them^[^ ^2^, ^68^, ^69^, ^70^ ^]^ because of the best multi‐material and multi‐color printing ability of it. Polyjet allows for easy and precise control to the mechanical properties of organ model by using photopolymers and resins (e.g., TangoPlus series form Stratasys) with various hardness and elasticity.^[^ ^64^ ^]^ These materials are mainly developed by relevant 3D printing companies, and few of them are prepared by researchers themselves.

Material extrusion and vat photopolymerization printing hard models can be visually impressive,^[^ ^71^ ^]^ but their rigidity limits the further application. For flexible materials, there is a limited variety available. Some commercial materials like thermoplastic elastomer (TPE), thermoplastic polyurethanes (TPU), and polyvinyl alcohol (PVA) filaments are soft materials that can be used in material extrusion, but their flexibility is far from that of real soft tissue. On the other hand, preparing soft materials manually always requires people to control some additional conditions, such as modifying the material system^[^ ^20^ ^]^ to improve the viscosity to help keep the shape of material during the printing process. Based on this, silicone and hydrogel can be used to print some simple blood vessels, trachea or prostheses.^[^ ^15^, ^37^ ^]^ Similar applications are more common in 3D bioprinting.^[^ ^72^ ^]^ Powder bed fusion and binder jetting techniques are used to a lesser extent. Although they have fast print speed,^[^ ^18^ ^]^ the printed models do not easily achieve good mechanical properties.^[^ ^73^ ^]^ These two techniques have been used in some studies to make hard trachea,^[^ ^74^ ^]^ heart^[^ ^75^, ^76^ ^]^ and other colorful models.^[^ ^73^, ^77^ ^]^

Taking Polyjet 3D printing technology as an example, the typical steps of direct 3D printing method are to first select the appropriate type of 3D printer according to the required accuracy and quality, and then select the material with appropriate physical properties from the materials available for 3D printer. Import the digital model file into the 3D printer for 3D printing, and enter the next post‐processing step after the printing is finished.

The characteristics of the direct 3D printing method are easy and convenient, especially less workflow, but the relative low cost FDM and binder jetting technology can only manufacture models with lower requirements, while the higher accuracy techniques like SLA and more comprehensive techniques like Polyjet use more expensive materials and devices with higher maintenance costs.^[^ ^18^ ^]^ Even if these conditions may be easily qualified in laboratories, there is still a constant demand to find ways to reduce costs for the extensive application of organ models in medical environment. Direct 3D printing method is almost suitable for making organ models of any structure. However, the noteworthy advantage of direct 3D printing is the competence to manufacture various tubular and channel structures with complex branches, and there are more alternatives for making blocky structures.

#### 3.2.2 Indirect 3D Printing Method

Indirect 3D printing method refers to the use of 3D printing to manufacture the parts for making the model, but not directly print the organ model itself. The most common is 3D printing a mold to solidify the liquid material to construct the organ model. Another indirect approach is the “sacrificial material method”, in which sacrificial objects are created by 3D printing. After being covered with other materials, these sacrificial structures can be melted or dissolved away to obtain the desired hollow structure or flow channel, often for making the vessels inside organs.^[^ ^78^, ^79^ ^]^

It is easier to 3D print molds than organ models because the forms are simpler. Various hard materials are available and any color is acceptable. The most basic desktop 3D printers are often sufficient. In the event of poor surface roughness of the mold caused by low accuracy of 3D printing, it can generally be settled by grinding or polishing process. Furthermore, extra coating is a considerable approach, such as applying some self‐leveling resin on the surface of the mold to make it smoother.^[^ ^80^ ^]^

Regarding the demoulding process, direct demoulding is a friendly method and produces less waste. The specially designed assembly structure of the mold can be disassembled and the model is taken out directly without breaking the mold. This places additional demands on previous workflows, including comprehensive CAD work and preparation of release agents. In contrast, the way to destroy the mold is particular about the choices of 3D printing technology and materials. Because the molds cannot be reused, which signifies the necessity of low‐cost, destructible materials, and fast 3D printing methods.

In terms of 3D printing, material extrusion, vat photopolymerization, powder bed fusion and binder jetting are all commonly used technologies. Material extrusion, especially the representative FDM technology has the dominant advantage in comparison with other methods, principally because of the low cost of materials and equipment with simple mechanical structure.^[^ ^81^ ^]^ It also has a fast printing speed on condition that pursuing less accuracy. FDM is quite capable of realizing various practices of indirect 3D printing method. The available materials range from PLA^[^ ^18^ ^]^ and ABS (acrylonitrile butadiene styrene) plastics which have excellent mechanical properties and are adequate for mold, and the water soluble PVA and limonene soluble HIPS (High impact polystyrene) which are suitable for sacrifice structures. It is noteworthy that the state of the PVA material can be different in different methods of creating organ models. Generally, one form of PVA is solid filament as a 3D printing material, and the other is hydrogel, which often requires special preparation and is used for casting. Wax is also a good sacrificial material. The cases of 3D printing wax for making organ models are relatively limited,^[^ ^82^ ^]^ but there are also ways to make molds by 3D printing models and then to cast wax,^[^ ^83^ ^]^ which is a combination of the 3D printing mold method and sacrificial material method.

In regard to vat photopolymerization, taking DLP as an illustration, is similarly a convenient technique to make molds. DLP has higher printing accuracy than FDM and produces smoother surface of the mold. Unfortunately, because of the lack of solubility for cured resins and the inevitable material residues on the surface of printed products, DLP method is generally not used for making sacrificial structures, and the printed mold requires adequate washing to avoid adverse effects on silicone curing.

Analogously, powder bed fusion and binder jetting methods are generally used to manufacture molds, but are less appropriate for printing sacrificial structures. In comparison with metals and ceramics, nylon^[^ ^84^ ^]^ or some plaster composites^[^ ^48^ ^]^ perform better in the field of organ models.

The following step is preparation of casting materials. A variety of efforts cover the exploration of silicone, polydimethylsiloxane (PDMS), agarose gel and some hydrogel.^[^ ^48^, ^85^, ^86^, ^87^, ^88^, ^89^ ^]^ For better simulating the characteristics of real tissues, the properties of materials need to be further controlled before use, embodied in the appropriate selection for the hardness of silicone, the addition of slacker additive into silicone solution,^[^ ^48^ ^]^ and the adjustment of ratio of components in hydrogel mixtures. Generally, silicone and PDMS are the easiest to use because of their convenience in storage, straightforward curing process, availability at any time, and cheapness. By comparison, the preparation and application of agarose gel and hydrogel have to undergo a more stringent process as well as special storage.^[^ ^48^, ^82^, ^90^ ^]^ However, when it comes to mimicking those particularly soft tissues, like the brain, the weaknesses can be covered up to some extent by its wide range of softness variability that other materials cannot achieve.^[^ ^91^, ^92^ ^]^

To illustrate the typical steps of indirect 3D printing method, take the FDM with silicone casting process as an understandable instance. The first is to select the appropriate 3D printer and material, such as a mid‐range grade desktop 3D printer and PLA filament material. Then make a mold through 3D printing, necessary surface treatment and assembly. Next, prepare the cast material by mixture and vacuum degassing of liquid silicone, and then pour it into the mold, waiting for the curing process, or accelerating curing by appropriate heating with attention to the control of temperature to avoid the thermal deformation of the mold. In general, the duration of curing process ranges from several hours to tens of hours according to type and quantity of silicone. After curing, carefully release the model and protect it from damage.

Unlike direct 3D printing method control the color of models directly by 3D printing, indirect method coloring the model through mixing pigments into materials in the stage of preparation or dyeing in the post‐processing step.^[^ ^48^ ^]^

Indirect 3D printing methods are characterized by lower costs for 3D printers and materials, but more complex workflow and more procedures for manual operation. On the one hand, manual operation can significantly save the cost of machinery manufacturing, but on the other hand, it will unavoidably increase the time cost and the error of the model, unless the operator has a high level of proficiency and skill. Generally, the indirect method is more suitable for manufacturing soft blocky structures, such as kidney and liver parenchyma. After all, making those soft materials that cannot be directly 3D printed play a role in organ models is the crucial advantage of indirect methods. For the fabrication of hollow tubular structures, such as bronchial, the indirect method remains some limitations such as the difficulty of designing an appropriate mold and the ununiformity of coating material on sacrificial structure, but some feasible and valuable attempts have been reported to improve the situation.^[^ ^93^ ^]^

#### 3.2.3 Combination of Direct and Indirect Methods

In virtue of the respective advantages of direct and indirect method, they can be combined for maximum effect. Maddox et al.^[^ ^88^ ^]^ used 3D printing to make a soft capsule and injected agarose gel solution into it, created a kidney model that subjectively felt‐like normal renal tissue. Ishii et al.^[^ ^94^ ^]^ directly 3D printed the blood vessels and shells of the liver, then 3D printed the mold and molded the liver parenchyma with soft polyurethane resin. Kuroda et al.^[^ ^10^ ^]^ used TPE to 3D print blood vessels and plaster to print mold, also molded liver parenchyma with soft polyurethane. Witowski et al.^[^ ^95^ ^]^ used PLA to 3D print blood vessels and tumors of the liver and print the mold, then cast with transparent silicone to make the liver parenchyma. Adams et al.^[^ ^82^ ^]^ directly 3D printed wax to make the collecting system of kidney, then printed photosensitive resin molds and casted with silicone, agarose gel and PDMS respectively to produce three different renal parenchyma, but it is noteworthy that they finally dissolved the wax in ethanol to remove the wax, therefore it can also be considered a completely indirect method.

The combination of direct and indirect method can improve the cost‐effectiveness, as each plays to its advantages. The selection is very personalized and requires not only the comprehension of characteristics of each 3D printing technology, but also the concrete analysis of specific scenarios. Besides, the careful comparison of printing resolution, available materials (including material color, texture, hardness, printability, and casting properties^[^ ^52^ ^]^) and printing time and cost of different methods are all the significant considerations for making any choice. Among these factors, for direct 3D printing method, higher manufacturing accuracy is often required, because it directly affects the accuracy of the model. It also requires better properties of materials, which means the materials need to meet both simulation performance and printability requirements. For indirect 3D printing method, although the requirement for manufacturing accuracy is lower, there are additional requirements for post‐processing. In terms of material properties, the requirements of materials for making molds are the lowest, while materials used for sacrificial structures need to be considered for their solubility, and materials for casting require constant exploration for new configuration methods to obtain appropriate properties.

To summarize, combining these factors to get the answer to the initial question, that is, in which aspects let 3D printing work.

### 3.3 Post‐Processing

After completing the 3D printing step, the physical model always needs to be post processed. The requests fall into two categories: 1) common post‐processing for most organ models consists of surface treatment, coloring and assembly; 2) special post‐processing for organ models with particular requirements, the major is sterilization.

Surface treatment is aimed at improving the surface smoothness of the model. Sandpaper, electric abrasive tools and chemical polishing solutions are commonly used to do the grinding and polishing work. But surface treatment is often not necessary for soft models in direct method, thanks to the vat photopolymerization technology based on liquid materials provide a well surface property, while the surface properties of soft models in indirect method are determined by the quality of the mold, and in general the related operations should be implemented in the mold printing phase. There are also ways that building hydrogel layers on the inner surface of the final models to reduce the surface roughness.^[^ ^79^ ^]^ On the other hand, not all organ models need to have high surface properties, the importance of simplifying processes and reducing costs is as much as improving the quality of models.

Coloring is an optional step because there are ways to control the color of the model in the previous manufacturing process. It is also feasible to use pigments for additional painting if the color of 3D printing product is not satisfactory.

As regards assembly, it is necessary for the models manufactured in multiple parts. Various kinds of glue are commonly used for bonding the parts, and some models are assembled by suturing^[^ ^96^ ^]^ or piecing^[^ ^94^ ^]^ together. Checking the model against the CAD data in the computer at any time during the assembly process can reduce the error.

Among the special post‐processing, sterilization operation allows the models to be used during the surgery or to prolong the preservative time. One of the common techniques is ethylene oxide sterilization,^[^ ^69^, ^94^ ^]^ which is one of the chemical ways. Other methods are hydrogen peroxide and peracetic acid sterilization. However, high temperature and radiation sterilization^[^ ^97^ ^]^ are less used because of the limitation of the thermal deformation properties of 3D printing materials.

### 3.4 Select the Method Based on the Demand

Organ models are used to realize many functions, from the models to replicate the anatomical structure of the real organ for visual experience, to the models for practical operation and repeated experiments. Models with different functions have great differences in the 3D printing process, and therefore need a clear classifications.

In most of the existing reports, organ models are classified as a direction of the application of 3D printing in the medical field with various names, such as “anatomical models”,^[^ ^98^ ^]^ “prototypes”,^[^ ^3^ ^]^ “replica,”^[^ ^79^ ^]^ or “phantoms”,^[^ ^48^, ^78^, ^82^, ^99^, ^100^, ^101^ ^]^ and play a role in various surgical operations. Some other reports have further subdivided organ models according to specific criteria. Christensen et al.^[^ ^58^ ^]^ classified the organ model into anatomical models and modified anatomical models, which mainly depends on whether the digital model is major changed or minor changed during the image processing steps. Pietrabissa et al.^[^ ^64^ ^]^ pointed out that the requests of organ models are usually the need for visual inspection and mechanical interaction, and explained the diverse requirements of them very well. Qiu et al.^[^ ^102^ ^]^ sorted organ models focusing on different materials used in making them, and gave a detailed description of each kind of material.

**Figure 5:**
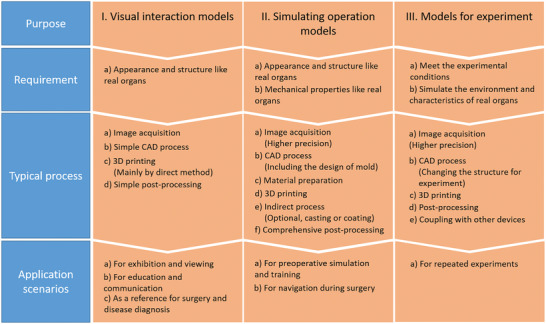
Different categories and requirements of organ models.

On the basis of current research and our definition of organ models, we classify the application requirements into three categories (**Figure** **5**): 1) models for visual interaction, 2) models for simulating operation, and 3) models for experiment.

The models for visual interaction only replicate the shape and anatomy of specific organs, but do not mimic the physical properties. People watch these models to enhance their understanding of organ structure. Some special designs^[^ ^66^, ^67^ ^]^ can not only make these models be 3D printed conveniently, but also expand the functions to meet the needs of surgical planning and teaching to some extent. It is not necessary for the models to be very precise, for example, making the blood vessels solid instead of hollow rarely affects the visual impressions. Then the size of models can be reduced considering the limitation of printing range of a 3D printer and the economization of materials, or be enlarged for better presentation of subtle structures. Last but not least, different colors and transparency for different structures guarantees a significant enhancement of visual effect. Almost all the 3D printing technology and materials used can well accomplish the task of “copying the shape”, but for these models with the lowest demand, the low‐cost method should be used whenever possible. FDM, SLA and Polyjet technologies can meet the requirements from the low to high level of visual interaction models through direct 3D printing method and hard materials of different colors. From the perspective of workflow, these models often require simple image acquisition, image processing and post‐processing.

A model created for simulating operation helps surgeons with simulation and training of surgical operation, or for preoperative planning and intraoperative navigation. It requires both to replicate the anatomical structure and to approach the “feeling” of real soft tissues, which means the similarity of biomechanical features. This kind of models often need to be made of soft materials and 3D printers with higher accuracy. Unquestionably, direct 3D printing method can be chosen when there are appropriate materials that accords with the features, while indirect method has more applicability for making such models. Indirect method controls the mechanical properties of materials conveniently and it is not limited by the printability. Moreover, sensors and real‐time feedback systems were applied to the training models in some studies,^[^ ^86^, ^103^, ^104^ ^]^ which provided more comprehensive information to enhance the training effect. The workflow of such models often contains all the steps. Whether using indirect methods or adding sensors will result in additional assignments at image processing steps, as well as more complex processes involved in 3D printing and post‐processing.

The last kind of models are mainly used for repeated experiments or for in vitro studies and device testing.^[^ ^105^ ^]^ A case in point is flow physiology research or hemodynamic evaluation.^[^ ^105^, ^106^ ^]^ These models are often coupled to some devices or placed specific implants, and input electric energy, fluid or other materials to perform experiments that are not feasible with human subjects and simulate the situation of real organs under experimental conditions. They can also be used for research of CT and MRI imaging functions.^[^ ^52^ ^]^ The requirements of these models remain the mimicking of biomechanical features, but the shape can be simplified as appropriate. The process of these models involves a similar workflow to the former ones. Meanwhile, considerations outside the manufacturing process such as experimental methods and equipment need more attention. Other ways like organ‐on‐chips system^[^ ^107^ ^]^ are likewise a significant organ simulation and experiment device, but fall outside the scope of our discussion.

## 4 Applications of 3D Printed Organ Models

**Figure 6:**
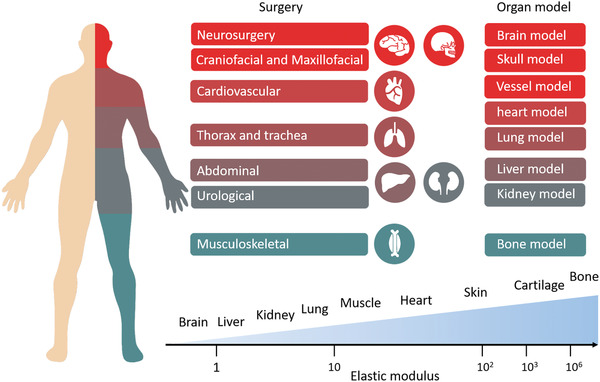
The application field of organ models. Different parts of human body correspond to different operations, and there are great differences in elastic modulus between real organs, which requires different organ models with different properties. Reproduced with permission. ^[^ ^108^ ^]^ Copyright 2021, American Chemical Society.

The emergence of 3D printing increases the possibilities for turning medical images of various organs into physical models. The efforts on constantly innovating the 3D printing technology and materials, and combining the diverse methods of creating organ models, continue to significantly make promotion in the clinical development and applications of organ models. Meanwhile, organ models play an important role in many kinds of major surgical procedures, as well as the communication and education outside of surgery. The concern is that models of different parts lay emphasis on different aspects. Thus, we focus on the application of organ models based on some parts of the human body including models for neurosurgery, cardiovascular, thorax and trachea, abdominal, and craniomaxillofacial as well as musculoskeletal, as shown in **Figure** **6**. In this section, we will review these applications and the significance of 3D printing in them.

### 4.1 Neurosurgery

Neurosurgical models mainly include the models of brain and spine. Faced with the intricate spatial location of nerves, vessels and sometimes involving the tumor, neurosurgeons often need to make accurate judgment before surgery and operate very carefully.^[^ ^109^ ^]^ Thus, the fabrication of brain models often involves varied selection of structures and application of multi materials. Any small mistakes can probably cause serious consequences.^[^ ^109^ ^]^ Although there are many noteworthy instances in other models,^[^ ^110^ ^]^ we focus on the efforts on making brain models.

**Figure 7:**
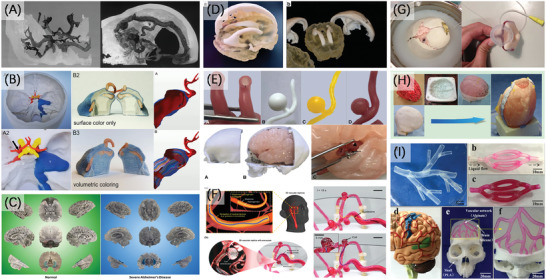
3D printing of organ models in neurosurgery. A) SLA 3D printed aneurysm and right temporal AVM models. Reproduced with permission.^[^ ^111^ ^]^ Copyright 1999, Elsevier. B) Binder jetting 3D printed colored chondrosarcoma model compared with 3D virtual model. Reproduced with permission.^[^ ^73^ ^]^ Copyright 2021, MyJoVE Corporation. C) 3D printed normal brain and severe (Severe‐1) Alzheimer's brain models. Reproduced under terms of the CreativeCommons CC‐BY license.^[^ ^112^ ^]^ Copyright 2017, The Authors. Published by Springer Nature. D) Multimodality imaging‐based 3D printed model of the neonatal head. Reproduced with permission.^[^ ^114^ ^]^ Copyright 2021, Springer Nature. E) The silicone models of vasculature lumen and brain. Reproduced under terms of the Creative Commons Attribution 4.0 International License.^[^ ^83^ ^]^ Copyright 2019, The Authors. Published by Springer Nature. F) 3D vascular replicas composed of elastomer–hydrogel skin multilayers and the simulation of endovascular intervention under optical images. Reproduced with permission.^[^ ^79^ ^]^ Copyright 2020, Wiley‐VCH. G) An endoscopic third ventriculostomy model in the cerebrospinal fluid–mimicking fluid and a neurosurgery model component containing the pons and basilar artery. Reproduced with permission.^[^ ^115^ ^]^ Copyright 2016, Elsevier. H) A cast brain model in the mock‐up skull together with the fluid. Reproduced under terms of the CreativeCommons CC‐BY license.^[^ ^91^ ^]^ Copyright 2016, The Authors. Published by Elsevier. I) Coaxial 3D printing of complex vessel‐like structures can be used for cerebral artery surgery simulator. Reproduced with permission.^[^ ^117^ ^]^ Copyright 2017, American Chemical Society.

In 1999, D'Urso et al.^[^ ^111^ ^]^ created the first 3D models for cerebral aneurysm and cerebral arteriovenous malformation (AVM) by SLA technology (**Figure** **7**A). Based on different needs, 3D printed neuroanatomical models often contain some specific structures of the brain, and others are simplified. A typical example is the skull model containing some vessels and tumors. The cavity of the skull represents the shape of the brain, with openings of different shapes designed on the skull to allow direct observation of internal structures, or some simulation. Plaster is an attractive material in these hard models because of its bone‐like properties.^[^ ^77^ ^]^ Although the plaster models made by binder jetting can have potential for breaking apart,^[^ ^73^ ^]^ the defects cannot obscure the virtues. Kosterhon et al.^[^ ^73^ ^]^ created two kinds of physical models containing different structures for patients with chondrosarcoma of the petrous apex by direct 3D printing method using multi‐color binder jetting technique, and used these models to surgical approach simulation and tumor resection (Figure 7B). They also pointed out the models were important tools in medical students and patient education. Marks et al.^[^ ^112^ ^]^ also made a contribution to the models for education, they directly 3D printed the models in some stages of Alzheimer's disease (Figure 7C). Some other hard materials are also used for direct 3D printing of such models, including thermoplastics and photopolymer resins. Scerrati et al.^[^ ^113^ ^]^ used FDM and SLA technology to print physical models of ABS filament and photoresin respectively to help surgical planning. They replicated the vessels thicker than 1 mm and discovered that the SLA model was relatively preferred by neurosurgeons. More recently, Wagner et al.^[^ ^114^ ^]^ showed a neonatal brain model based on multimodality imaging data and was printed with multi materials by Polyjet technique. The model can help with perioperative management (Figure 7D).

Regarding soft models, indirect 3D printing method comes in handy. Nagassa et al.^[^ ^83^ ^]^ combined two types of indirect methods to make the model of middle cerebral artery aneurysms (Figure 7E). They created the mold using mold making silicone rubber based on the original structure 3D printed in photopolymer resin. Then they cast water‐soluble wax as sacrificial structure, applied silicone on it and dissolved the wax to gain a vessel lumen. Moreover, they changed the sensitivity of an aneurysmal vascular wall by controlling the layers of silicone coated on the wax. Similarly, Lim et al.^[^ ^79^ ^]^ showed an indirect method for making vascular models composed of elastomer–hydrogel skin multilayers (Figure 7F). A dip coating method based on a supporting liquid was developed previously^[^ ^93^ ^]^ and in this way the 3D printed ABS sacrificial structure was uniformly coated with a thick layer of PDMS. A hydrogel skin was then built at the interface with the inner surface of PDMS. The multilayers of different materials made the inner surface of models smoother and addressed the shortcomings of 3D printing mold or sacrificial structure with high surface roughness.

The combination of indirect methods has also been used to create brain parenchyma, such as Cheung et al.^[^ ^115^ ^]^ applied a similar method to create brain parenchyma with hollow ventricle structure, and provided a realistic dynamic environment for the model by using mimic liquid that was composed of red pigment and milk (Figure 7G). In addition to silicone, hydrogels are also concerned due to their biocompatibility. The typical example is the composite hydrogel of PVA and PHY (phytagel), which shows excellent properties of mimicking soft tissue. Forte et al.^[^ ^91^ ^]^ developed a composite hydrogel to create brain models in indirect methods, and was capable of reproducing the brain shift phenomenon and brain tissue response to indentation and palpation (Figure 7H). Furthermore, Tan et al.^[^ ^116^ ^]^ made a comparison between composite hydrogel brain model and porcine brain tissue samples, and indicated that the material composition of 2.5 wt% PVA and 1.1 wt% PHY best matched the properties of porcine brain. Gao et al.^[^ ^117^ ^]^ have explored the direct 3D printing of hydrogel materials, and printed vascular structures with multilevel fluidic channels, which demonstrated the ability of hydrogels to construct tissue engineering structures. Based on their previous work,^[^ ^118^ ^]^ a cerebral artery surgery simulator was developed (Figure 7I). Although bioprinting and tissue engineering are not in the scope of our discussion, the way in which brain structure was built in their work is still valuable and noteworthy for the fabrication of organ models.

Examples that are just tips of the iceberg also illustrate the expanding possibilities of the combination utilization of hard and soft materials in brain models brought about by 3D printing technologies, satisfying the demands of mimicking diverse structures from skull to brain parenchyma with different hardness, which is almost impossible for traditional manufacturing methods.

### 4.2 Cardiovascular

Cardiovascular field is one of the most common application fields of medical 3D printing.^[^ ^52^ ^]^ 3D printing and bioprinting technologies provide important help for the treatment of congenital heart disease,^[^ ^119^, ^120^, ^121^ ^]^ aortic aneurysm,^[^ ^122^, ^123^ ^]^ cardiac tumor^[^ ^124^ ^]^ and other cardiovascular diseases, and have been introduced comprehensively in some studies.^[^ ^125^, ^126^ ^]^ As far as organ models are concerned, the common ones are the models of complete heart or parts of it,^[^ ^4^ ^]^ as well as the models for some vascular diseases. In terms of imaging, there are more technologies to choose from. In addition to CT and MRI, the most commonly used method is echocardiography.^[^ ^18^, ^125^, ^126^, ^127^ ^]^ The structure of heart is more complex than that of blood vessel, and the construction of blood vessel models are mostly similar to those methods described in neurosurgical models. Therefore, we pay more attention to the work of heart models in the category of anatomical models, and select some vascular models for the discussion of experimental models.

**Figure 8:**
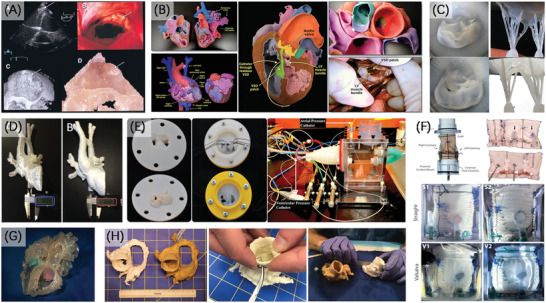
3D printing of organ models in cardiovascular. A) SLA 3D printed model of flail posterior leaflet. Reproduced with permission.^[^ ^128^ ^]^ Copyright 1999, American College of Cardiology. B) 3D printed colored heart models of patients of different ages and conditions. Reproduced with permission.^[^ ^130^ ^]^ Copyright 2017, American College of Cardiology Foundation. C) Two valve models fixed in the simulator for surgical training of annuloplasty, neo‐chordae and chordae‐loop implantation. Reproduced with permission.^[^ ^131^ ^]^ Copyright 2019, Springer Nature. D) 3D printed rigid and flexible models of aortic arch hypoplasia. Reproduced with permission.^[^ ^133^ ^]^ Copyright 2015, Wiley‐VCH. E) Silicone mitral valve model cast through 3D printed solid valve‐flange and incorporated into pulse‐duplicator system. Reproduced with permission.^[^ ^134^ ^]^ Copyright 2016, Elsevier. F) 3D printed left heart model simulator components for comparing the biomechanics of two conduits. Reproduced with permission.^[^ ^135^ ^]^ Copyright 2018, Elsevier. G) 3D printed heart models with translucent material, the Amplatzer device in the atrial septum is visualized. Reproduced with permission.^[^ ^137^ ^]^ Copyright 2016, Springer Nature. H) Physical measurements of 3D printed cardiac models and cadaveric hearts. Reproduced under terms of the CreativeCommons CC‐BY license.^[^ ^139^ ^]^ Copyright 2019, The Authors. Published by Springer Nature.

Binder et al.^[^ ^128^ ^]^ first applied echocardiography data with SLA technology to fabricate cardiac structures (**Figure** **8**A). Izzo et al.^[^ ^129^ ^]^ chose materials with different properties to mimic the soft vascular tissue and hard calcified structures of mitral valve. The heart model was printed by Polyjet technology and used for performing a mock procedure and optimizing the transapical surgical approach. Anwar et al.^[^ ^130^ ^]^ used material jetting technology to create heart models for patients across a spectrum of age with congenital heart disease (Figure 8B). These colorful models can distinctly show a variety of pathologies and help for surgical procedures and education.

Establishing a standardized production process for indirect 3D printing methods can effectively make up for the error caused by manual operation, and ensure the accuracy of the recapitulation of soft models. Engelhardt et al.^[^ ^131^ ^]^ produced silicone mitral valve models through a standardized process including FDM printing mold using transesophageal echocardiography (TEE) data and casting of silicone (Figure 8C). Particularly, the lower shell was made of water‐soluble PVA material so that the complex structure was then dissolved to avoid the difficulty of disassembly. In addition to the training and patient‐individualized rehearsal, the models were integrated into a flow simulator to perform hemodynamic evaluation.^[^ ^132^ ^]^

Similarly, in the past few years there have been numerous repetitive experiments and simulations using cardiovascular models. Valverde et al.^[^ ^133^ ^]^ 3D printed models of aortic arch hypoplasia with two different rigid using polylactic acid polymers, for improving the cases of stent migration and other complications in stenting (Figure 8D). The models were verified to be accurate and allowed unlimited simulation of different interventional strategies to determine the optimal stent length and release site. Mashari et al.^[^ ^134^ ^]^ also applied TEE data and indirect 3D printing method to make a silicone model based on the 3D printed rigid model, and performed hemodynamic testing (Figure 8E). It improved the situation that the discrepancies and discordance between transvalvular gradients and hemodynamically calculated and planimetered areas, and enhanced the understanding of postrepair hemodynamics.

Appropriate models are also conducive to graft testing. Paulsen et al.^[^ ^135^ ^]^ 3D printed a left heart model as a simulator including a valve‐sparing root replacement model and a physiologic coronary circulation, and mounted two different conduits into it (Figure 8F). Through the hemodynamic, echocardiographic, and high‐speed videometric data, they found the significant difference in the biomechanics of the two conduits, which might affect the clinical outcomes. For investigating the static and dynamic mechanical properties of the 3D printing materials under physiologically relevant loading, Stepniak et al.^[^ ^136^ ^]^ used FDM, SLA and Polyjet technologies 3D printed a hollow coronary artery network for use as an anthropomorphic plaque phantom, and established the mechanical and CT attenuation properties of materials. The model can be used in studies of the effect of various parameters on CT number measurements of lipid‐rich plaque and enable the design of optimal imaging protocol.

Furthermore, integrating multiple imaging strategies can magnify their strengths and make the data more appropriate for 3D printing, otherwise individual imaging modalities might not provide adequate visualization for the required structure, which is especially important for complex diseases (Figure 8G).^[^ ^137^ ^]^ Equally significant is the selection of suitable 3D printing technologies. Applying multiple 3D printing methods in the same model can clearly reflect the differences of different methods. Birbara et al.^[^ ^138^ ^]^ created heart models employing CT and 3D echocardiography data and four common 3D printing methods including SLA, FDM, SLS and polyjet. They demonstrated the applicability of 3D modelling and printing to different imaging modalities and focus on the discrepancies in the precision of models made by different methods. Through quantitative comparison, the results showed that SLA technology was the most precise. Odeh et al.^[^ ^139^ ^]^ also aimed at the accuracy of the model. They selected the cadaveric hearts for CT scanning to get the image data and 3D printed heart models in flexible white filament material. Various methods including physical measurements, digital photographic measurements and some other ways were used to verify the accuracy of 3D printed anatomical models (Figure 8H). The verification of part accuracy was one component of a quality assurance (QA), and a noteworthy point was that the quality of the model was always constrained by the quality of the imaging. Meanwhile, they gave the possible errors and measuring methods in every step from image acquisition to 3D printing model.

Although the cardiovascular system does not involve a hard structure like bone, the wide range of materials available in 3D printing makes the cardiovascular models still well reflect the property differences between different structures.

### 4.3 Thorax and Trachea

The organ models of thorax generally refer to the models of tissue and structure in thorax. Compared with the repetitive experiments mainly manifested as the simulation of hemodynamics in cardiovascular models, the thorax model is more applied for diverse imaging technology evaluation and dosimetry measurements.^[^ ^140^, ^141^ ^]^ As one of models for experiment, imaging phantoms are widely used in the test and optimization of imaging devices, avoiding the radiation burden on human body.^[^ ^52^, ^142^ ^]^ Moreover, 3D printed personalized model based on the clinical situation of specific patients is always required whether for imaging or the assistant of surgical procedures.

**Figure 9:**
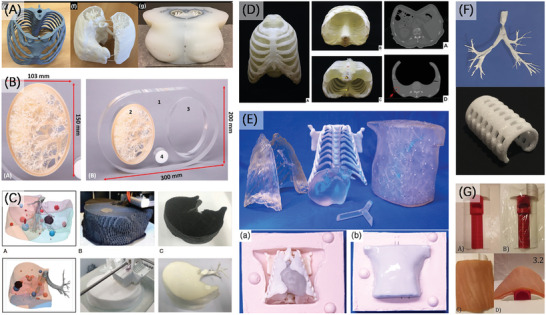
3D printing of organ models of thorax and trachea. A) 3D printed model of bone structures and lung structures including airways, blood vessels, and tumors. Reproduced with permission.^[^ ^142^ ^]^ Copyright 2017, American Association of Physicists in Medicine. Published by Wiley‐VCH. B) MJM 3D printed lung vessel model in a PMMA chest phantom container. Reproduced with permission.^[^ ^100^ ^]^ Copyright 2018, Elsevier. C) FDM 3D printed CT phantoms of lung. Reproduced under terms of the CreativeCommons CC‐BY license.^[^ ^143^ ^]^ Copyright 2020, The Authors. Published by Wolters Kluwer Health. D) 3D printed thorax model consisting of the skeletal integument embedded in the transparent 3D printed body. Reproduced under terms of the CreativeCommons CC‐BY license.^[^ ^99^ ^]^ Copyright 2020, The Authors. Published by Frontiers Media S.A. E) 3D printed resin molds and models of neonate's thorax with optical properties. Reproduced under terms of the CreativeCommons CC‐BY license.^[^ ^144^ ^]^ Copyright 2020, The Authors. Published by SPIE. F) 3D printed nylon model of a pediatric airway with tracheal stenosis and external tracheal splint made of PCL. Reproduced with permission.^[^ ^145^ ^]^ Copyright 2020, Elsevier. G) FDM 3D printed low‐cost trachea model covered with artificial skin. Reproduced under terms of the CreativeCommons CC‐BY license.^[^ ^146^ ^]^ Copyright 2017, The Authors. Published by Cureus Inc.

Hazelaar et al.^[^ ^142^ ^]^ showed a life‐size phantom assembled from bony, lung structures and lung tumors, which were printed in different materials and methods (**Figure** **9**A). The construction method of bone and soft tissue was similar to the previous organ models, while the trachea and vessels were printed by SLS technology using nylon material. Because nylon has high strength with a density that is comparable to biological materials. The model can be used to evaluate x‐ray‐based imaging quality and positional verification techniques for radiotherapy. Hernandez‐Giron et al.^[^ ^100^ ^]^ applied the multi jet modeling (MJM) technique, a kind of material jetting method, selected the vessel tree as a part of human lungs anatomy to print the model with a minimum structure of 0.25 mm size, which fell in the range of diameters of order 6 vessels and below the resolution of usual commercial CT systems (≈0.5 mm) (Figure 9B). The printed vessel tree was inserted in an elliptical holder to form a general thorax surrogate model, and was used for the clinically relevant and reproducible measurement of CT image quality.

In addition to making the shape of the model be comparable to the real organ, the material properties or manufacturing parameters of the model can be further modified to reproduce not only the geometry but also the radiodensity. Zhang et al.^[^ ^101^ ^]^ filled a mixture of CaCO_3_, MgO, agarose, NaCl, pearl powder and silica gel in the model printed with ABS material, created an individualized anthropomorphic lung model with tissue‐equivalent radiation attenuation properties and can be used for radiation dose verification. Similarly the use of low‐cost method, Hong et al.^[^ ^143^ ^]^ used FDM technique to fabricate lung models and investigated the Hounsfield Units (HU) for models with different materials (including PLA, TPU, and ABS) and different filling density through CT scanning (Figure 9C). They also randomly added various kinds of nodules to the lung models for evaluating the size‐accuracy of the quantification software, as well as for the precise calibration of CT intensity. More recently, Hatamikia et al.^[^ ^99^ ^]^ developed a novel radiopaque mixture constituting of epoxy, polypropylene and bone meal powder (Figure 9D). The mixture was filled into the skeletal integument of the Polyjet 3D printed model through a specific mold, and thus the model was able to mimic realistic HUs of different skeletal components of the thorax. Furthermore, different tumors and lesions could be added in the modifiable modular model. But the authors also mentioned some problems that manual operation and the limitation of specifications of 3D printer brought some errors to the model as well as some fragile structure caused by material properties. Therefore, the characteristics of 3D printing technology and materials should be taken into consideration in the CAD processing steps.

Other experiments, such as gas sensing, monitoring and evaluation of gas spectroscopy, are also unique to the lung model compared with other organ models. Tobo et al.^[^ ^144^ ^]^ showed a multi structure tissue model of the thorax of a neonate by indirect 3D printing method, which can be used for gas in scattering media absorption spectroscopy (GASMAS) measurements of oxygen content within the lung cavity (Figure 9E). In particular, they developed a specific mixture constituting of ink and spheres to match the optical properties of the different organs.

Regarding the thoracic and tracheal surgery, 3D printing models as anatomical models play a role to help with the surgical procedures (Figure 9F).^[^ ^145^ ^]^ The anatomically accurate trachea models can improve the emergency cricothyroidotomy simulation. Doucet et al.^[^ ^146^ ^]^ utilized FDM technique and an entry‐level desktop 3D printer to print a low cost model of the cartilage within the trachea (Figure 9G). The tracheal model cost only $1.70 Canadian dollars (CAD) but was flexibility and durability. Wu et al.^[^ ^147^ ^]^ chose SLA technique to print the model of pleural tumors and adjacent structures for preoperative simulation, surgical rehearsal, and surgical planning of tumor resection. The repair conformal titanium plates were designed matching patient's specific defect based on the 3D printed model, which reduced the bleeding, postoperative complications and pain. Richard et al.^[^ ^148^ ^]^ applied Polyjet technique to 3D print airway model used for surgical planning of high‐grade laryngotracheal stenosis, which verified the feasibility of 3D printing model in the treatment of airway diseases.

Coupling 3D printing to appropriate design of structures and material composition offers tremendous opportunities for organ models to mimic real organs from mechanical properties, optical properties to radiation imaging properties, and therefore to create various functional organ models for more level applications.

### 4.4 Abdominal and Urological

The abdominal and urological organ models include the models of stomach, intestine, liver, gallbladder, kidney, and other related organs and tissues. The application of 3D printing in these organ diseases has been systematically reviewed before.^[^ ^64^ ^]^ Therefore, here we will not discuss all of them, but focus on the advances in kidney and liver models, in which 3D printing technology is most commonly applied.^[^ ^64^ ^]^

#### 4.4.1 Kidney

Renal transplantation is a common renal surgery.^[^ ^149^ ^]^ The growing demand of available organs and the strict requirement of safety of donors and recipients^[^ ^70^ ^]^ make renal transplantation surgery face more severe challenges.

**Figure 10:**
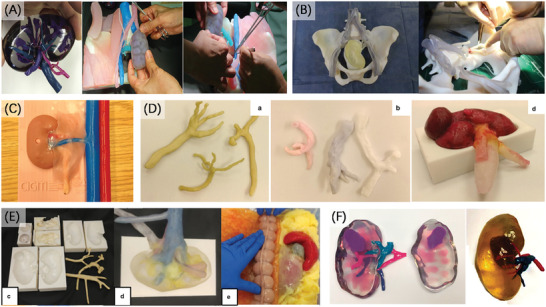
3D printing of kidney models. A) 3D printed donor kidney models, graft and pelvic cavity model used for preoperative surgical simulation. Reproduced with permission.^[^ ^70^ ^]^ Copyright 2015, Elsevier. B) 3D printed prosthetic cradles of kidney model and the addition of deceased donor vessels to the cradles. Reproduced under terms of the CreativeCommons CC‐BY license.^[^ ^154^ ^]^ Copyright 2018, The Authors. Published by Springer Nature. C) A pediatric pyeloplasty simulator and a kidney model using silicone to mimic fat. Reproduced with permission.^[^ ^155^ ^]^ Copyright 2016, Elsevier. D) 3D printed PVA structure of the renal artery, renal vein, and calyx for casting kidney model used for rehearsal in progress in a mock operating room. Reproduced with permission.^[^ ^158^ ^]^ Copyright 2021, Springer Nature. E) A kidney model cast by 3D printed injection molds and used for full procedural rehearsal on daVinci robot. Reproduced with permission.^[^ ^153^ ^]^ Copyright 2019, Springer Nature. F) 3D printed models of renal cell carcinoma, renal cancer and prostate cancer. Left‐hand side: Reproduced with permission.^[^ ^161^ ^]^ Copyright 2016, Elsevier. Right‐hand side: Reproduced with permission.^162^ Copyright 2017, Springer Nature.

Kusaka et al.^[^ ^70^ ^]^ first applied 3D printed organ model in renal transplantation (**Figure** **10**A). They used CT data and Polyjet technology to directly print kidney models from multiple materials, which were used to build simulation and navigation programs for kidney transplantation surgery. The iliac vein of the model was stained with blue dye to make a better distinction of the structure. More recently, Claflin et al.^[^ ^150^ ^]^ developed an interactive and cost‐effective training model for kidney transplantation. The models of the donor kidney and the recipient abdomen were printed with PLA and ABS filament respectively, with a total cost of $178. This training model aimed at the training of specific surgical skills instead of the replication of a specific patient's anatomy.

Another advantage of simulation using 3D printed models is the feasibility to dock the models with surgical robots to perform more comprehensive training, on the basis of helping surgeons for the conventional planning and simulation of various kinds of kidney surgery.^[^ ^8^, ^151^, ^152^ ^]^ Some robot assisted operations, such as robot‐assisted partial nephrectomy (RAPN), are challenging because of their significant learning curve.^[^ ^96^ ^]^ The application of 3D printed organ models makes up for the current scarcity of the training model for these operations (Figure 10B).^[^ ^96^, ^153^, ^154^ ^]^ Monda et al.^[^ ^96^ ^]^ referred to the indirect method from Cheung et al.,^[^ ^155^ ^]^ (Figure 10C) 3D printed the mold and mixed silicone and deadener material in the mass ratio of 9:4 to cast a kidney model, which provided a training tool for robot‐assisted laparoscopic partial nephrectomy.

In recent years, Ghazi et al.^[^ ^156^, ^157^, ^158^ ^]^ have made a series of explorations on the simulation of real kidney with soft materials (Figure 10D). Among these efforts, Melnyk et al.^[^ ^153^ ^]^ used CT data, indirect 3D printing method, and chose PLA filament to print the mold while PVA filament to print sacrificial structure (Figure 10E). Furthermore, PVA powder was mixed with water to develop a biocompatible hydrogel to cast the final kidney model. The model was used for the full procedural RAPN simulation. In their studies, 7% PVA at three freeze–thaw cycles was considered to be the best formula to mimic the properties of fresh porcine kidney tissue.

Similarly, for the training of robot‐assisted kidney transplantation (RAKT), Saba et al.^[^ ^149^ ^]^ applied various PVA hydrogel formulations to cast the donor kidney models. Then they created a model of recipient pelvic including a bony pelvis, pelvic musculature, iliac arteries and veins, and bladder. Artificial blood was used to simulate bleeding during surgery. The total cost of the two models was $95, which was also cost‐effective.

Based on the docking to other devices, 3D printed models are sometimes combined with virtual reality (VR) and augmented reality (AR) technologies^[^ ^159^, ^160^ ^]^ to help disease treatment from more dimensions. Physical models remain unique advantages as compared with the other approaches.

In the past few years, Wake et al.^[^ ^161^, ^162^ ^]^ have done a series of work on organ models of kidney and prostate tumors (Figure 10F). They compared the 3D printing model with AR and VR, and found that the 3D printing model was most helpful for patients to understand the characteristics of organs, disease and surgery, thanks to the realistic haptic feedback provided by physical models, which is impossible in other methods.^[^ ^163^ ^]^

#### 4.4.2 Liver

Hepatobiliary surgery often requires high‐level surgical techniques and 3D anatomy knowledge.^[^ ^94^ ^]^ The application of 3D printed models can enhance anatomical understanding of people and meanwhile help the patient education.^[^ ^164^, ^165^ ^]^

**Figure 11:**
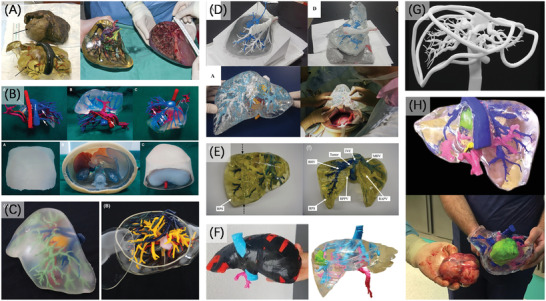
3D printing of liver models. A) Preoperatively 3D printed liver and right lobe models compared with actual organs. Reproduced with permission.^[^ ^166^ ^]^ Copyright 2013, American Association for the Study of Liver Diseases. Published by Wiley‐VCH. B) 3D printed liver model and abdominal cavity model allowed for precise management of the middle hepatic vein. Reproduced with permission.^[^ ^167^ ^]^ Copyright 2019, American Association for the Study of Liver Diseases. Published by Wiley‐VCH. C) Liver models made of soft polyurethane resin with a hard shell used for intraoperative navigation. Reproduced with permission.^[^ ^94^ ^]^ Copyright 2020, Wiley‐VCH. D) 3D printed vessel system and plaster template to manufacture the liver model for preoperative simulation and intraoperative navigation. Reproduced with permission.^[^ ^10^ ^]^ Copyright 2019, Springer Nature. E) 3D printed model of metachronous liver metastasis from sigmoid colon cancer used for planning partition line. Reproduced with permission.^[^ ^169^ ^]^ Copyright 2017, Royal Australasian College of Surgeons. Published by Wiley‐VCH. F) The assembly of 3D printed molds and casting of silicone liver model. Reproduced under terms of the CreativeCommons CC‐BY license.^[^ ^80^ ^]^ Copyright 2017, The Authors. Published by Springer Nature. G) A novel 3D printed liver model made up of frames representing the main structure of the liver. Reproduced with permission.^[^ ^66^ ^]^ Copyright 2016, Springer Japan. H) Life‐sized liver models developed by standard 3D printing and silicone casting. Reproduced under terms of the CreativeCommons CC‐BY license.^[^ ^95^ ^]^ Copyright 2019, The Authors. Published by Springer Nature.

The need for liver transplantation surgery is similar to kidney transplantation. Zein et al.^[^ ^166^ ^]^ used contrast‐enhanced CT and MRI data and Polyjet method, directly printed the first complete 3D‐printed livers with multiple materials (**Figure** **11**A). Model was used for making optimal surgical plans in living donor liver transplantation (LDLT). In particular, they chose appropriate coded color to stain different vascular structure, thus optimizing the visualization of the model.

The materials in indirect 3D printing methods to fabricate liver models remain abundant options. Wang et al.^[^ ^167^ ^]^ focused on pediatric LDLT, selected photopolymerization technique to directly print the liver model, while utilized a tissue‐like feel silicone to cast the abdominal model (Figure 11B). They pointed that physical liver models contributed to reducing the risk of large‐for‐size syndrome and graft reduction in small infants. More recently, Ishii et al.^[^ ^94^ ^]^ and Kuroda et al.^[^ ^10^ ^]^ made liver models with soft and transparent parenchyma by casting polyurethane. The former model (Figure 11C) had a transparent and hard shell printed by photopolymerization method, and was used for intraoperative navigation of liver transplantation. The latter model (Figure 11D) was based on the previous work of the authors,^[^ ^69^ ^]^ adding the process of casting the liver parenchyma through 3D printed plaster mold, and for use in planning and simulation of LDLT.

The vascular structure in 3D printed liver model is a vital content. Clear visualization of key areas of vessels is helpful for vascular reconstruction in complex liver surgery. Huber et al.^[^ ^168^ ^]^ fabricated the liver parenchyma by polyurethane rubber material and material injectors, with ABS filament printed gallbladder and other structures. The application of this liver model in complex vascular reconstruction facilitated the planning of surgery.

Other common applications of 3D printed liver models include preoperative planning and intraoperative navigation model (Figure 11E,F,G),^[^ ^66^, ^69^, ^80^, ^169^ ^]^ which can be a complete liver structure or contain partial of it, for hepatectomy and resection of hepatocellular carcinoma, as well as the model for multimodal image‐guided liver therapy research.^[^ ^78^ ^]^ Witowski et al.^[^ ^95^, ^170^ ^]^ utilized CT scanning to study the accuracy of 3D printed liver model applied in laparoscopic liver resection, and quantitatively evaluated the influence of liver model on the surgery (Figure 11H). The approaches involved in their studies are low‐cost. Nonetheless, the models were sufficiently accurate, and can help to properly plan the extent of liver resection.

The principles of 3D printing technology give people the farthest freedom in the manufacture and use of organ models, and are almost not limited by the process and environment. All of these show tremendous potential to create functional organ models.

### 4.5 Craniomaxillofacial and Musculoskeletal

Different from the various models introduced above, both craniomaxillofacial models and musculoskeletal models often involve a large number of hard structures to represent the bone. Although it is easy to 3D print hard structures most of the time, there are still many aspects worthy of attention, such as the application of low‐cost methods and the simulation of bone properties.

#### 4.5.1 Craniofacial and Maxillofacial

Although craniomaxillofacial may not be the most commonly used aspect of 3D printed organ models, it is one of the earliest applications of 3D printing in medicine.^[^ ^52^, ^57^ ^]^

The concept of craniomaxillofacial involves pathologic conditions of the head and neck,^[^ ^52^ ^]^ in which 3D printing technologies are often used to create physical models, prostheses and implants^[^ ^11^ ^]^ based on these bone structures to aid with the craniomaxillofacial plastic surgery, restoration and reconstruction.^[^ ^46^, ^59^, ^171^, ^172^ ^]^ Therefore, the concrete examples of 3D printed models in these applications are likely to involve ophthalmic models,^[^ ^173^, ^174^ ^]^ otorhinolaryngologic models,^[^ ^49^ ^]^ and dental models.^[^ ^175^, ^176^ ^]^ The fabrication of implants is a significant application of 3D printing in craniofacial reconstruction. Although 3D printing of Implants is beyond the scope of this review, several studies have demonstrated the advantages of 3D printing in creating various customized implants.^[^ ^11^, ^177^ ^]^ Generally, the concept of a prosthesis is different from the organ model, but it is noteworthy that the manufacturing process and properties requirements of prosthesis are very similar to the organ model. Thus, we will also select some cases of 3D printing nasal and auricular prosthesis for brief introduction but will not discuss them in detail.

**Figure 12:**
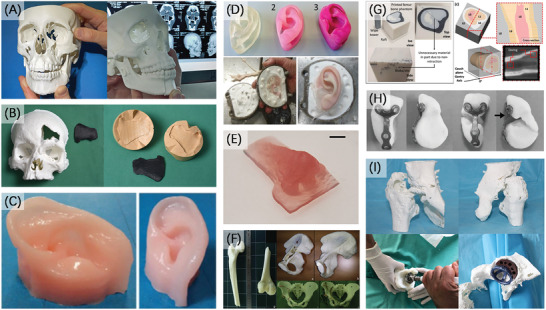
3D printing of organ models in craniomaxillofacial and musculoskeletal. A) FDM 3D printed low‐cost haptic model of left zygomatic fracture in clinical practice as a support for classical tomodensitometry. Reproduced with permission.^[^ ^179^ ^]^ Copyright 2019, Wiley‐VCH. B) FDM 3D printed model of the forehead (white) and cast implant template (black). Reproduced with permission.^[^ ^180^ ^]^ Copyright 2017, Wolters Kluwer Health. C) Silicone auricular prosthesis fabricated by SPPC method. Reproduced with permission.^[^ ^118^ ^]^ Copyright 2014, Springer Nature. D) Original ear models printed by Polyjet technology and final auricular prosthesis cast by silicone. Reproduced with permission.^[^ ^181^ ^]^ Copyright 2018, Taylor & Francis. E) DIW 3D printed exemplary silicones nasal model with complex 3D structures. Reproduced with permission.^[^ ^37^ ^]^ Copyright 2019, American Chemical Society. F) FDM 3D printed bone models of different fractures. Reproduced with permission.^[^ ^187^ ^]^ Copyright 2018, the Japanese Orthopaedic Association. Published by Elsevier. G) Patient‐specific femur model printed by interlace deposition method of standard PLA and Fe‐PLA filament, and the workflow. Reproduced with permission.^[^ ^188^ ^]^ Copyright 2020, Elsevier. H) 3D printed scaphoid models with a prebent plate in situ. Reproduced with permission.^[^ ^190^ ^]^ Copyright 2018, SAGE Publications. I) 3D printed life‐size hemipelvis model for a series of tests and to help make decisions. Reproduced with permission.^[^ ^191^ ^]^ Copyright 2019, Oxford University Press.

Mankovich et al.^[^ ^178^ ^]^ in 1994 first applied 3D printed custom CT‐based auricle model for skull reconstruction. Grunert et al.^[^ ^103^ ^]^ 3D printed a head model with plaster as base material and realized an electric circuit in order to provide feedback for the training of otologic intervention. Benefiting from the situation that the model is mainly composed of a large number of hard bone structures, the low‐cost direct 3D printing method is widely applied. The available materials like thermoplastic and plaster are both cost‐effective and easy to use. Nicot et al.^[^ ^179^ ^]^ chose FDM technology and ABS filament to print a low‐cost craniofacial trauma model that played a role in the oral and maxillofacial surgery teaching program (**Figure** **12**A). The whole cost less than US$10, with a complete set weighing less than 100 g that allowed easily transported. Msallem et al.^[^ ^180^ ^]^ also applied FDM method but chose PLA filament to print a model of a craniofacial defect with involvement of the supraorbital rim (Figure 12B). They designed and printed a customized implant based on this model. The whole process of fabricating the model and implant was simple without the requirement of advanced devices, which was beneficial to the application in the setting with limited resources.

Applying the prosthesis for restoring missing facial parts is a low risk and effective treatment.^[^ ^181^ ^]^ The typical examples are the uses of nasal and auricular prosthesis, which need to accurately replicate the structure of real organs and mimic the texture and color of skin. These requirements have a lot in common with organ models. He et al.^[^ ^118^ ^]^ used the Scanning Printing Polishing Casting (SPPC) method to create silicone auricular prosthesis (Figure 12C). The SPPC was a representative of low‐cost indirect 3D printing method, which improved the time‐consuming and costly situation of conventional manual process. Similarly, Mohammed et al.^[^ ^181^ ^]^ implemented a more complex indirect method, including Polyjet 3D printing original models and multi phases of manufacturing of molds and models (Figure 12D). The final auricular prosthesis was cast by silicone and used for facial restoration. Through their process, the cost was reduced by one‐third and production time was reduced ≈5 h compared with the traditional method.

With regard to directly 3D printing silicone, Unkovskiy et al.^[^ ^182^ ^]^ printed the silicone nasal prosthesis as the interim postsurgical appliance because it was mainly limited by the permission of medical use of 3D printable silicone material. They also showed a feasible digital workflow from defect acquisition to prosthesis, with the reminder that the manual finishing of prosthesis can effectively improve the esthetic outcome. Furthermore, Zhou et al.^[^ ^37^ ^]^ made efforts on the modifying of material system to enhance the printability of silicone. They showed the silicone nasal prosthesis printed by DIW method, but was not applied in practical treatment yet (Figure 12E). Other flexible materials in the direct method can also be used as prosthesis manufacturing materials. Like the work that mentioned above, Nuseir et al.^[^ ^183^ ^]^ also gave a complete digital workflow to construct a nasal prosthesis. They chose Polyjet technology printed flexible material to create a nasal prosthesis and achieved the effect of restoring facial aesthetics. Gao et al.^[^ ^15^ ^]^ developed a kind of GelMA/Nanoclay ink to 3D print a bionic ear, which expanded the potential for customized treatment of tissue defects.

#### 4.5.2 Musculoskeletal

There have been a variety of other organ models containing the bone structures in the previous cases. Nevertheless, 3D printing of bone models of specific parts remains useful in various orthopedic operations including diagnosis of orthopedic diseases, training and planning of surgery.^[^ ^184^, ^185^ ^]^

Cherkasskiy et al.^[^ ^186^ ^]^ used FDM technique and ABS filament to 3D print proximal femur model for the planning and practice of triplane proximal femoral osteotomy. They set a 20% honeycomb fill pattern of printing parameters in order to mimic the cut resistance of real bone. Kim et al.^[^ ^187^ ^]^ showed the ABS filament printed acetabular models analogously (Figure 12F). The model aided in planning the optimal positioning of a reduction clamp and the trajectory of screws. Tino et al.^[^ ^188^ ^]^ proposed an inexpensive interlace deposition method to 3D print bone‐equivalent models (Figure 12G). The materials they chose were PLA and iron‐reinforced PLA (Fe‐PLA) filaments, and the CT imaging attenuations were controlled through the dual‐extrusion printing in an interlacing pattern with variable layer thickness, which showed the potential for personalized dosimetry in radiotherapy. Gregory et al.^[^ ^189^ ^]^ evaluated the potential of 3D printed models in preoperative planning from the perspective of the feasibility of disease classification. In their studies, eight models of different patients with fractures of the distal humerus were created and assessed by orthopedic surgeons with different seniority. The moderate agreement provided by the experiment indicated the feasibility of using 3D printed models for disease assessment. Ten Berg et al.^[^ ^190^ ^]^ utilized thermoplastic powder to print models of the proximal and distal nonunion fragments and the intact scaphoid (Figure 12H). Based on the thermoplastic powder, they additionally expressed that medical‐grade polyamide, polycarbonate or resin were all recommended materials to print the low‐cost organ models.

Revision hip arthroplasty or severe reconstructive procedures often require high skill level surgical crew and adequate planning. Tserovski et al.^[^ ^191^ ^]^ 3D printed the hemipelvis model for preoperative surgical drill with position simulation of revision hip surgery (Figure 12I). However, they pointed out that 3D printing added additional technical requirements like advanced computer skills.

Just like exploring the 3D printed organ models of soft materials that mimic the soft tissue properties, the 3D printing of bone based models makes advances in approach of bone properties, which is equally valuable for the progress of 3D printing and bioprinting.

## 5 Discussion

In the above section, we reviewed and summarized the recent progresses in 3D printing of organ models. The concepts of 3D printing of organ models contain the 3D printing technologies and the characteristics of organ models, which correspond to methods and products respectively. The selection of methods determines the quality and effect of products, and therefore affects the role and application of products. It is necessary to discuss “what is a good organ model?” when we talk about 3D printing of organ models. In other words, what kind of quality and effect is considered good for organ models? We want to share some of our understandings and views about this topic.

In the development of organ models, numerous researchers are making efforts to make organ models more “like” real organs, not only from the visual or tactile, but also from specific biomechanical properties. However, is the model that more closely resembles a real organ the better? We might as well take the 3D printing technologies as a point. When we evaluate the quality of 3D printing methods in specific applications, no matter how high‐precision and high‐printing speed that the method is, the cost is a criterion that must be considered. A typical set of examples are the FDM and Polyjet technologies. Obviously, the main reason that makes FDM popular and competitive around the world is not its high printing accuracy and speed, but its low cost and simple mechanical structure of devices. People can easily turn their ideas into reality through FDM technology in their office or even at home. In contrast, Polyjet as another common technology, which is famous for its powerful functions, but provides challenges for users who are not scientific researchers because of its high requirements for routine maintenance and the additional requirements for operation due to the possible toxicity of resin materials to human body. Only for the above two examples of 3D printing technologies, we are not able to absolutely evaluate which is the better technology. The same is true with organ models made by 3D printing.

For 3D printed organ models, we can easily list some general evaluation criteria, including the material properties, appearance, manufacturing accuracy, manufacturing speed, manufacturing cost, and even the ease of design and use.^[^ ^4^ ^]^ It is readily comprehensible that some of the standards restrict each other. For example, the pursuit of high precision and speed often leads to higher cost and more design difficulty. Moreover, high precision models are not always necessary for application. Most existing studies have mentioned that cost is one of the major limitations of 3D printing of organ models,^[^ ^3^, ^4^, ^49^, ^57^, ^59^, ^64^, ^119^, ^126^, ^192^, ^193^, ^194^, ^195^ ^]^ including the manufacturing cost and time, especially the time required for the whole process, which often leads to the model not applicable for emergency surgery.^[^ ^196^ ^]^ Furthermore, the limitations of the organ model also include: 1) Model accuracy,^[^ ^52^, ^119^, ^192^, ^194^, ^197^ ^]^ in other words, the errors in each step of the model may lead to serious consequences in the operation. It remains to be discussed who is responsible for these problems.^[^ ^195^ ^]^ A feasible way is to explain the errors that may be included in the model carefully to the surgeons before handing the model over to them, and assist the surgeons to judge and act according to their experience. 2) The lack of materials to simulate the real tissue properties,^[^ ^3^, ^18^, ^57^, ^119^, ^193^, ^195^, ^197^, ^198^ ^]^ and the limited mimicking ability of organ models.^[^ ^4^, ^102^ ^]^ 3) The lack of large‐scale research and application cases to verify the function of organ models.^[^ ^49^, ^57^, ^192^, ^193^ ^]^ 3) The lack of unified standards for organ models.^[^ ^119^, ^192^ ^]^ 4) The potential environmental protection,^[^ ^119^ ^]^ regulatory,^[^ ^3^, ^45^, ^58^, ^195^, ^199^ ^]^ and ethical^[^ ^195^, ^199^ ^]^ problems and risks.

Therefore, before discussing “what is a good organ model”, there are some questions worth thinking about, such as “is it necessary to require a particularly high accuracy of an organ model?”,^[^ ^18^ ^]^ “is it easy for the environment (especially in the hospital) to have the required conditions and afford the corresponding manufacturing costs?”, “Does surgeons really need organ models for all kinds of surgery?”. That is why some of the studies we reviewed emphasized that their methods are low‐cost, and the produced organ models can also meet the needs of users. However, we don't mean that 3D printing of organ models should blindly reduce costs. On the one hand, for research, it is worthwhile to realize continuous breakthroughs in functions, even if it may face high costs. On the other hand, for the application, how to make high‐performance products more convenient in daily applications has become the most important concern. Functions and cost‐effectiveness are to application feasibility as two legs are to a person, not a single one of these conditions can be dispensed with. From our experience, compared with the requirements for higher performance of 3D printing technologies, the requirements for more careful post‐processing of 3D printing products are more likely to give consideration to both product performance and cost‐effectiveness, just like the methods mentioned above such as polishing the 3D printed molds^[^ ^118^ ^]^ or coating special material layers on the models.^[^ ^79^ ^]^ Through these approaches, only a low‐cost 3D printing technique is needed to make a high‐performance product.

In general, we do not want to restrain the direction of organ models in “make them like real organs”, but rather let a hundred flowers bloom. Organ models with integrated and extended functions are equally attractive. In any case, the advantages of 3D printing used in organ models are obvious, including the manufacturing process is far more convenient than traditional methods, and the possibility of realizing complex structures. Different organ models play a role in different surgical operations and are helpful for preoperative planning, simulation and intraoperative navigation, thus saving the operation time and reducing the operation risk. Most importantly, the additional time spent on 3D printing organ models is invaluable for saving time in surgery to save lives.^[^ ^59^ ^]^ Besides surgery, organ models can also be used for repetitive experiments, medical training, doctor‐patient communication and patient education, and promote the communication and cooperation between medicine and engineering.^[^ ^4^, ^18^, ^57^, ^59^, ^119^, ^192^, ^197^ ^]^ All of these provide direction and opportunity for the future development of organ models and 3D printing in medicine.

## 6 Future Perspectives

**Figure 13:**
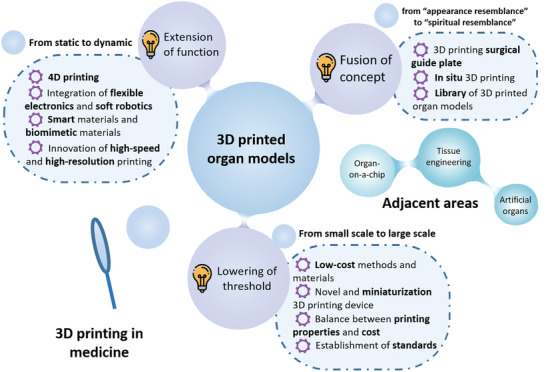
The future development of 3D printing of organ models.

From CAD processing of medical image data to 3D printing, the combination of modern digital design and manufacturing technology has provided great possibilities for creating the organ models and expanding their functions, and boosted 3D printing technologies in medical field. With the continuous innovation of technologies and materials for 3D printing organ models, various new approaches have not only gradually met the increasing requirements, but also broken through the existing limitations of organ models. Although, in many cases, the organ model is regarded as only a “model”, it remains the potential to be given deeper concepts (**Figure** **13**).

First, from static to dynamic. The question is, how to further expand the function of organ models? In this review, the current application requirements of organ models have been divided into three categories, but there is still much room for further discussion. Most of the organ models are static,^[^ ^102^ ^]^ while a few dynamic models sometimes appear in the relevant experiments represented by the research of flow physiology. Making the organ models from “static” to “dynamic” is a feasible direction, which will realize more application modalities in the future. Fortunately, the thriving 3D printing technologies for soft materials are promising choices to make “dynamic” organ models. In addition to printing the soft organ models directly, these approaches are capable of integrating the functions and devices of flexible electronics and soft robotics into 3D printed organ models, thereby realizing the integrated manufacturing of comprehensive functional organ models. More importantly, other noteworthy concepts are 4D printing^[^ ^200^, ^201^ ^]^ and self‐assembly.^[^ ^202^ ^]^ In 4D printing the printed materials will change with time or external conditions such as the 4D printing of high‐performance thermal‐responsive swellable materials,^[^ ^203^ ^]^ while self‐assembly utilizes cells to construct vascularization and functionalization structures. These “dynamic” effects may be beyond the scope of current organ models, but it is still worth learning. The application of these techniques in organ models will further expand the dynamic function of the model from the dimension of structural change.

Second, from small scale to large scale application. How to make organ models more convenient for manufacturing and application in medical environment? Creating organ models in the hospital and in the laboratory or 3D printing company are two different contrasting choices. Although it is convenient to produce organ models through laboratories or 3D printing companies outside hospitals, the immediate problem is that the additional transportation and storage process may damage some soft and fragile organ models. A helpful alternative is setting up a small studio staffed by doctors and engineers in the hospital, and utilizing the small‐sized 3D printing devices is enough. Regarding the manufacturing cost, it involves a lot of interdisciplinary research. There is no doubt that 3D printing will continue to break through in the future development and the cost of mature 3D printing technologies will be further reduced, but the progress of 3D printing technologies is not our focus. For manufacturing organ models, 3D printing is more like a tool, and whether the tool can produce excellent results often depends on how people use it. Irrespective of how the future development of 3D printing is, it is always significant to choose appropriate methods, equipment and materials based on clear use requirements within the available range of 3D printing technologies. On the other hand, the future development of 3D printing organ models is not only closely related to the progress of 3D printing technology, but also goes hand in hand with the improvement of printing materials, design tools, process algorithms, and post‐processing techniques.^[^ ^204^ ^]^ Furthermore, there is still a lack of a large number of randomized controlled trials to evaluate the effectiveness of organ models.^[^ ^49^, ^57^, ^192^, ^193^ ^]^ One of the reasons is that organ models are still difficult to be widely applied. Therefore, manufacturing models conveniently in medical settings will be useful for further verification of effectiveness of organ models, and establishing the whole cycle investigation of organ models from image to 3D printing to application effect. In turn, these studies can promote the establishment of the standardization of 3D printing organ models or even, on a large level, 3D printing of medical devices and other products.

Finally, from “appearance resemblance” to “spiritual resemblance”. Let 3D printing of organ models and related research fields complement each other. Human beings have developed various organs with different ingenious structures over millions of years of evolution. The recent efforts made by numerous scientists to “imitate” these organs have formed many research fields, including 3D bioprinting,^[^ ^205^, ^206^, ^207^ ^]^ tissue engineering,^[^ ^202^ ^]^ organ‐on‐a‐chip^[^ ^208^ ^]^ or 3D printed microfluidic devices,^[^ ^209^ ^]^ and even the 3D printing of biomimetic materials and structures.^[^ ^210^ ^]^ These studies are closely associated with organ models, while the difference is whether cells are involved or not. The developments of these similar fields are not independent, but on mutual promotion and common progress with others. The technologies, materials and methods are related and worth learning from each other. Like a 3D printed vascular structures can be used as a simulating model without cells and as a tissue engineering product with cells.^[^ ^117^ ^]^ It is also a common strategy to construct vessel networks in 3D bioprinting by using fugitive/sacrificial materials.^[^ ^204^ ^]^ From blood vessels to the whole heart, Dvir et al.^[^ ^211^ ^]^ also demonstrated the feasibility of personalized hydrogels to print heart with major blood vessels. Certainly, the conditions of 3D bioprinting are much more strict and complex than 3D printing organ models, which is not just adding cells into the printing process. With that being said, we believe that the current challenges they face (such as the complexity of the fabricated structure, the accuracy of the printing, and the speed of the process)^[^ ^204^ ^]^ and future directions can be discussed together. In the farther future, the innovation of 3D printing will constantly expand the potential of 3D bioprinting, and therefore the mutual enhancement of physical organ models and in vitro tissue models^[^ ^212^, ^213^ ^]^ will be more significant. 3D bioartificial organs^[^ ^204^, ^214^, ^215^ ^]^ with complete organ functions will be the ultimate embodiment of the integration and development of these fields. Although there is still a long way to go, this is the inevitable pathway for organ models to go from “appearance resemblance” (having the same physical shape as real organs) to “spiritual resemblance” (having the same biological functions as real organs) that worth people making unremitting efforts.

## 7 Conclusion

In summary, 3D printing of organ models is an essential direction of the application of 3D printing in medicine. 3D printing technologies enable people to create various physical organ models with complex anatomical structures conveniently and quickly. With the continuous innovation of manufacturing methods and materials, there has been a lot of advancement in the accuracy and the tissue simulation performance of organ models. In this review, we have summarized the utilization of 3D printing in fabricating organ models for different using requirements and emphasized the role that 3D printing can play in each stage of the manufacturing process. The applications of organ models in corresponding surgery are also be introduced. We anticipate this review could help readers create organ models more conveniently, especially in the medical settings instead of in the industrial conditions to select the appropriate methods and materials, and achieve organ models with more functions. In the interdisciplinary era, the convergence of advanced technologies in similar fields is inevitable, which will promote the development of organ model to a higher dimension and bring revolution to people's life and health in the future.

## Conflict of Interest

The authors declare no conflict of interest.
